# Freestanding Emergency Department Entry and Market‐level Spending on Emergency Care

**DOI:** 10.1111/acem.13848

**Published:** 2019-10-22

**Authors:** Vivian Ho, Yingying Xu, Murtaza Akhter

**Affiliations:** ^1^ Rice University's Baker Institute Houston TX; ^2^ Department of Economics Rice University Houston TX; ^3^ Department of Medicine Baylor College of Medicine Houston TX; ^4^ University of Arizona College of Medicine–Phoenix, Maricopa Medical Center Phoenix AZ

## Abstract

**Background:**

Freestanding emergency departments (FrEDs) could reduce wait times in overcrowded emergency departments (EDs), but they might also increase usage and overall spending for emergency care. We investigate the relationship between the number of FrEDs entering a local market and overall spending on emergency care.

**Methods:**

We accessed data from Arizona, Florida, North Carolina, and Texas in Blue Cross Blue Shield Axis; a limited data set of deidentified insurance data claims that we linked to Public Use Microdata Area (PUMA) data from the American Community Survey; and lists of licensed FrEDs from state agencies. Regression analysis was used to estimate the association between changes in the number of FrEDs in 495 PUMAs and total spending on emergency care, out‐of‐pocket spending, utilization, and price per visit from January 2013 to December 2017. Final estimates came from a PUMA‐level fixed‐effects model, with controls for state, quarter, and PUMA‐level demographics.

**Results:**

Entry of an additional FrED in a PUMA was associated with a 3.6 percentage point (pp; CI = 2.4 to 4.9) increase in emergency provider reimbursement per insured beneficiary in Texas, Florida, and North Carolina. There was no change in spending (2.5 pp; CI = −8.2 to 3.1) associated with a FrED's entry in Arizona. Entry of an additional FrED was associated with a 0.18 (CI = 0.12 to 0.23) increase in the number of emergency care visits per 100 enrollees in Texas, Florida, and Arizona. In contrast, entry of another FrED was not associated with a change in utilization (−0.03; CI =  −0.09 to 0.02) in North Carolina. Estimated out‐of‐pocket payments for emergency care increased 3.6 pp (CI = 2.5 to 4.8) with the entry of a FrED in Texas, Florida, and Arizona, but declined by 15.3 pp (CI = −26.8 to −3.7) in North Carolina.

**Conclusions:**

Rather than functioning as substitutes for hospital‐based EDs, FrEDs have increased local market spending on emergency care in three of four states’ markets where they have entered. State policy makers and researchers should carefully track spending and utilization of emergency care as FrEDs disseminate to better understand their potential health benefits and cost implications for patients.

The annual number of emergency department (ED) visits rose by 18.4% between 2006 and 2014, accompanied by an increase in the average age and number of comorbidities among ED patients.^1^ Correspondingly, the share of civilian, noninstitutionalized persons’ health expenditures devoted to ED care rose from 3.6% to 4.4% during this time period.^2^ Providers in some states have met this increased demand by opening freestanding emergency departments (FrEDs).

FrEDs deliver emergency care in a facility that is physically separate from an acute care hospital. Some FrEDs are owned by a parent hospital and are referred to as a “satellite” to that hospital, while other FrEDs have no such hospital affiliation.^3^ Texas, Ohio, and Colorado were documented as having the most FrEDs in 2015 (181, 34, and 24, respectively), but 360 were located across 30 states.^4^ Proponents of FrEDs claim that these facilities can relieve the burden of overcrowded waiting rooms in hospital‐based EDs, while promptly caring for patients in more convenient locations.^5^, ^6^

However, critics of FrEDs argue that the facilities increase spending, because they serve as supplements to traditional EDs rather than substitutes, delivering care that could be provided in alternative lower cost settings.^7^ Furthermore, policy researchers are concerned that existing private insurer and Medicare payment policies are encouraging providers to shift services from lower paying settings such as urgent care centers and physicians’ offices to higher paying settings such as FrEDs.^8^, ^9^

Similar to hospital EDs, FrEDs charge a facility fee for each visit. Retail and urgent care clinics or doctors’ offices do not charge this fee.^10^, ^11^ The facility fee originated under the Medicare program and was intended to compensate hospitals for the operational expenses of maintaining an outpatient facility, but facility fees are also charged to patients with private insurance coverage.^12^, ^13^ A recent study found that prices for patients with similar diagnoses were 10 times higher at FrEDs in Texas compared to urgent care clinics, with the majority of the price difference attributable to the facility fee charged by FrEDs. The average price for a visit to a FrED in 2015 was $2,199, which was comparable to a hospital‐based ED visit, with 82% of that price being the facility fee.^14^

Although past studies have documented the rapid growth of FrEDs in multiple states, there is only limited information about how entry of FrEDs has influenced overall spending on EDs and how much changes in spending are attributed to shifts in price versus utilization. In this study, we analyze ED claims data from four U.S. states to examine the association between the entry of FrEDs in local markets and total ED spending, utilization, price, and out‐of‐pocket spending. The analysis provides useful insights on how much FrEDs serve as a substitute for care provided at existing hospital‐based EDs versus contributing to a rise in overall use of EDs. This information is valuable to state regulators and public and private insurers who must consider regulations and reimbursement policies for FrEDs.

## Methods

### Data

#### Blue Cross Blue Shield Axis Claims Data

We accessed the Blue Cross Blue Shield (BCBS) Axis limited data set, which is estimated to contain claims for 175 million active and inactive (previously enrolled) commercially insured members between 2012 and 2018.^15^ The BCBS Axis data are a limited data set under HIPPA privacy rules, because it excludes 16 categories of direct identifiers and is used for research purposes without obtaining prior authorization from patients.^16^ The BCBS companies in BCBS Axis are licensees of BCBS Association, an association of independent, locally operated Blue Cross and Blue Shield companies.

We restricted the analysis to claims from Arizona, Florida, North Carolina, and Texas. We chose these states, because they all experienced entry of FrEDs during the sample period and because BCBS companies have the largest market share in each of these states, which increased the number of claims available to analyze.^17^ These states also vary in population size, presence of Certificate of Need (CON) regulations, whether they impose policies specific to FrEDs, and other legal requirements (see Data Supplement S1, Table S1, available as supporting information in the online version of this paper, which is available at http://onlinelibrary.wiley.com/doi/10.1111/acem.13848/full).^18^ We analyzed claims from 2013 to 2017, because these were the only years available in BCBS Axis when we began our analysis. This secondary analysis of facility and professional claims for EDs was accessed through a secure data portal. The institutional review board of Rice University considered this study exempt from review.

Claims data for ED visits were identified using the National Committee for Quality Assurance's methodology. The NCQA counts any claim as emergency related if it contains a CPT code of 99281‐5, which are procedure codes for ED visits for evaluation and management of a patient, or if the claim contains a UB revenue code of 0450‐2, 0459, or 0981—codes for hospital services delivered in the ED. The NCQA also counts claims with a place of service code of 23 (emergency room–hospital) and one of more than 5,000 ED‐related procedure codes as emergency claims.^19^ Any claims data satisfying these criteria for the years 2013 through 2017 were drawn for the sample. No 2013 claims data were available for Arizona; no claims data for Florida were available between 2015 Q3 and 2017 Q2.

For each claim within the sample we recorded the “total allowed amount,” which is the combined amount the provider should receive from the insurer and out of pocket from the patient. We subtracted from this number the “paid amount,” which is the amount the insurer paid to the provider, to estimate the amount billed to the patient to be paid out of pocket

#### FrED Entry and Markets

Our approach for identifying FrEDs in Texas was described previously.^20^ We relied on licensing data from the Texas Department of State Health Services, as well as Internet searches, e‐mails, and phone calls. In Arizona, Florida, and North Carolina, only hospitals are allowed to open FrEDs. The names, addresses, and effective dates of operation for FrEDs were obtained from the state health department in each of these states.

Using the address of each FrED, we determined which Public Use Microdata Area (PUMA) it was located in. Developed by the Census Bureau, PUMAs are geographic units constructed by combining census tracts (or counties in sparsely populated areas) with geographical contiguity. Each PUMA must contain over 100,000 residents, and the majority of PUMAs contain 100,000 to 200,000 residents.^20^ For example, Texas has 212 PUMAs. A total of 38 of those are located in Harris County, which covers 1,778 square miles and includes Houston, the fourth largest city in the U.S. In contrast, the “Rio Grande COG & Permian Basin Regional Planning Commission” PUMA in west Texas spans 36,606 square miles and contains 14 counties.

Defining local markets using PUMAs is consistent with the economics literature, which finds that population density plays a central role in firm entry decisions.^21^, ^22^ PUMAs are much smaller in geographic size than the more well‐known Hospital Service Areas defined by the Dartmouth Atlas.^23^ For example, the HSA for Houston contains 38 hospitals and is divided into roughly 50 PUMAs. Market sizes at the PUMA level are more likely to reflect the decision facing consumers considering the use of EDs—visiting a nearby FrED with little or no wait time for care or traveling slightly farther to a hospital ED where roughly one‐third of patients waited an hour or more for care.^24^

#### Constructing PUMA‐level Variables

Emergency claims data were attributed to PUMAs based on the zip code of residence of the patient on the date of treatment. Claims data were then aggregated to obtain the total amount of expenditures on EDs by PUMA, year, and quarter. ED spending per enrollee was calculated by summing the allowed amount in the facility and professional claims in each PUMA and dividing by the number of enrollees (whether or not they had any claims) in the PUMA. Out‐of‐pocket ED spending per enrollee was calculated in a similar manner.

We subdivided spending on EDs into utilization versus price. The number of emergency visits per 100 enrollees was calculated by dividing the number of unique facility claims on each date for each enrollee by the number of enrollees/100 in each PUMA. An estimate for the “price” of each visit was obtained by dividing ED spending for each PUMA by the number of facility claims.

PUMA‐level data on the percentage of the population with any insurance along with the percentage covered by Medicare and Medicaid; median household income; the percentage of the population Hispanic, black, or with a high school diploma; and population count by year were obtained from the American Community Survey. Based on previous studies, these variables were hypothesized to influence demand for ED services among insured persons.^25^, ^26^

Although we are only measuring factors associated with ED use, previous studies have found that patients with different coverage types use EDs at different rates. Higher or lower propensity to utilize EDs by insurance type can influence the patient load and wait time at hospital EDs, which may affect decisions by consumers on whether and where to seek care in an ED when new facilities become available.

### Data Analyses

Descriptive statistics were used to compare the number of FrEDs by PUMA at the beginning of the sample (2013 Q1) and the end (2017 Q4) for Arizona, Florida, North Carolina, and Texas. Insured status and other sociodemographic characteristics included in the American Community Survey, the mean volume of enrollees by PUMA, total PUMA population, and mean values of the dependent variables in the regressions by PUMA for these time periods are listed by state. We graphed mean ED spending per enrollee by quarter and state.

We then applied regression analysis to test whether the entry of one or more FrEDs to a PUMA was associated with a change in ED spending per enrollee by year and quarter, adjusting for other factors that might influence spending. We also tested for an association between FrED entry and the number of ED visits per enrollee and the price per visit. The unit of analysis for the regressions is a PUMA during the quarter of a given year. The explanatory variable of interest was a continuous measure of the number of FrEDs in a PUMA in a given year and quarter. We included an interaction of state indicator variables with FrED counts to test whether the association between facility entry and ED spending differed across states. In cases where the state interaction term was statistically significant, the change in the dependent variable associated with FrED entry was the linear combination (sum) of the coefficients of the number of FrEDs and the state interaction term.

The multiple measures of insured status and socioeconomic status that were considered as explanatory variables were highly correlated. To avoid potential problems of multicollinearity, variables with a variance inflation factor greater than 2.5 were excluded from the regressions.^27^ The excluded variables were the percentage covered by Medicaid, median household income, the percentage of the population Hispanic, and population count in each PUMA.

The regressions included fixed effects for each of the 20 quarters in the sample, PUMA fixed effects, and interaction effects of a linear time trend with each PUMA fixed effect. Inclusion of these fixed effects and interactions controls for potential systematic trends in ED spending across PUMAs that may have coincided with the entry of FrEDs. With the inclusion of PUMA fixed effects, the FrED explanatory variables measure the association between within‐PUMA changes in the number of FrEDs and ED spending.

The regressions involving per‐capita spending and price were estimated using a generalized linear model (GLM) with a log link.^28^ Expenditures and price data commonly follow a skewed rather than normal distribution, which suggests that the relation between the explanatory variables and these monetary values is better modeled with a log distribution. The GLM allows one to estimate this relation while avoiding biases that can result from estimates derived from ordinary least squares with the log of spending or price as the dependent variable.^28^ The regressions were estimated using Stata 15.1. The glm command in STATA allows one to adjust the standard errors to account for correlation in the error terms within PUMAs, and the regressions were weighted by the number of enrollees in each PUMA.

### Sensitivity Analysis

Census experts form PUMAs based on population counts rather than land area. Therefore, one might be concerned that PUMAs may not be the correct unit of analysis in rural areas. As a sensitivity analysis, we limited the sample to PUMAs where 100% of the land area was located in a Metropolitan Statistical Area as defined by the census. Major insurers are trying to control rising ED spending by denying reimbursement for ED visits that they deem unnecessary.^29^ We estimate an additional regression with the insurer's amount paid to the provider per enrollee as the dependent variable to determine whether the results are consistent with those obtained with spending per enrollee.

## Results

Table 1 presents descriptive statistics on the 495 PUMAs by state at the beginning and end of the sample period. Despite similar population numbers across PUMAs, FrED entry was more widespread in Texas than in the other three states. By 2017 Q4, 74% of PUMAs in Texas had at least one FrED, while only 28% of PUMAs in Arizona, 22% of PUMAs in Florida and 14% of PUMAs in North Carolina had one or more FrEDs.

**Table 1:** Characteristics of PUMAs by State and Quarter

	Arizona		Florida		North Carolina		Texas	
	2014 Q1^a^	2017 Q4	2013 Q1	2017 Q4	2013 Q1	2017 Q4	2013 Q1	2017 Q4
0 FrEDs	51	39	141	118	71	67	151	56
1 FrED	2	14	10	28	7	10	39	54
2+ FrEDs	1	1	0	5	0	1	22	102
% Insured	87.8	91.5	82.4	90.2	87.1	92.3	80.4	86.0
% Medicare	20.2	21.6	24.1	26.3	20.7	22.7	15.9	17.5
% Medicaid	19.5	22.3	16.4	17.7	17.0	17.9	16.7	16.6
Median household income ($)	53,918	58,524	48,974	55,110	49,137	55,004	56,790	62,051
% Hispanic	26.2	26.9	22.1	23.3	6.4	6.9	35.0	36.1
% Black	4.8	5.2	15.4	14.8	21.1	20.6	11.8	11.5
% w/High school diploma^b^	19.0	18.8	23.0	22.5	21.1	20.7	19.0	19.2
Total population	124,657	129,099	129,489	137,458	126,257	130,586	124,756	132,391
No. of BCBS enrollees	13,939	16,845	20,444	25,166	27,357	28,522	23,768	22,741
Spending per Enrollee ($)	51.65	57.90	116.5	163.8	53.23	79.81	132.18	215.92
Utilization per 100 enrollees	3.32	3.16	4.93	5.83	2.42	2.42	6.27	7.83
Price per Visit ($)	1,568	1,838	2,370	2,832	2,314	3,620	2,150	2,834
Out‐of‐pocket spending per enrollee	19.57	14.02	30.27	26.21	16.06	16.57	54.19	71.78
No. of PUMAs	54		151		78		212	

FrED = freestanding ED; PUMA = Public Use Microdata Area.

a Claims data for Arizona were available beginning in 2014.

b Highest educational degree achieved.

**Figure 1:**
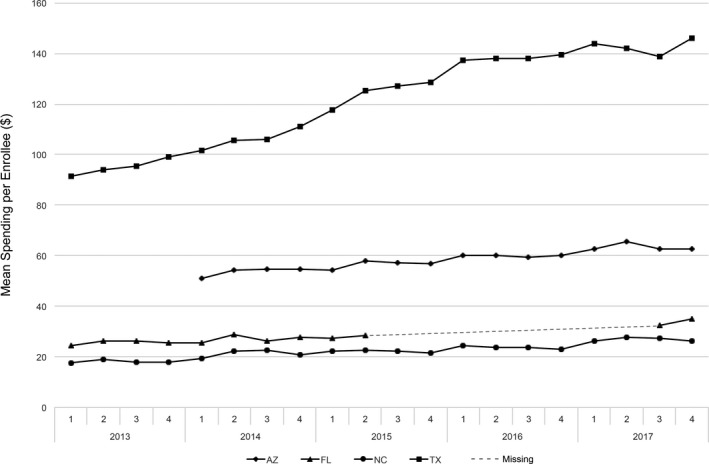
Mean ED spending per BCBS enrollee by state and quarter. BCBS = Blue Cross Blue Shield.

Figure 1 illustrates trends in ED spending per enrollee by state during the sample period. All states displayed a general upward trend in ED spending per enrollee, with Texas showing the steepest increase. Florida and North Carolina had the lowest spending per enrollee, while spending per enrollee was substantially higher in Texas.

**Figure 2:**
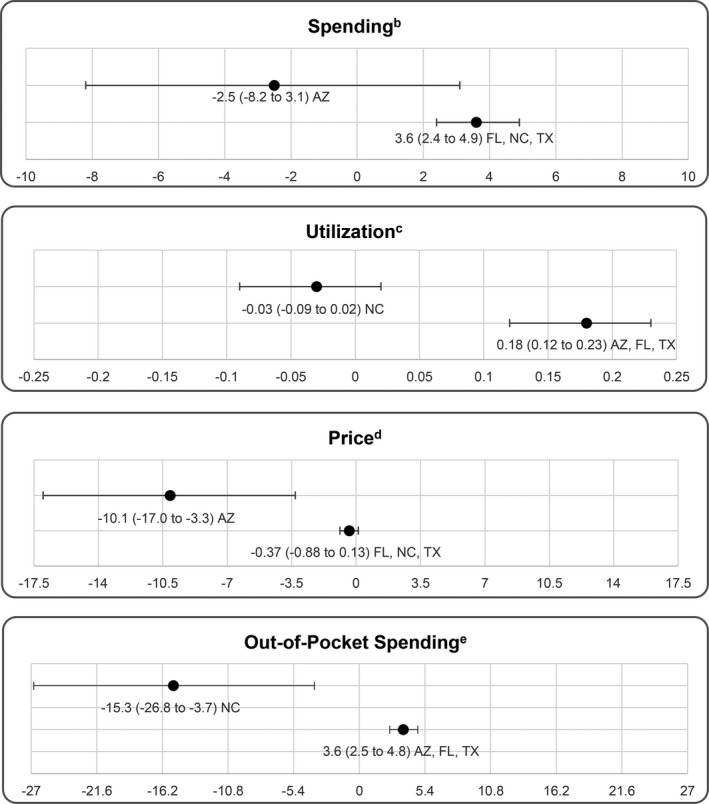
Adjusted estimates of the relation between entry of a freestanding ED in a local market and percentage change in per‐capita spending, utilization, price, and out‐of‐pocket expense.^a^ ^a^The reported estimates apply to entry of one additional FrED in a PUMA. Each additional FrED is associated with an additional change in the dependent variable by the same amount. ^b^Spending refers to the combined amount spent by insurers and patients on emergency care per insured enrollee. ^c^The number of emergency care visits per 100 insured beneficiaries. ^d^Emergency care spending divided by the number of emergency care visits in each PUMA. ^e^Out‐of‐pocket spending refers to the amount of the payment for emergency care services per enrollee that the patient is responsible for. FrED = freestanding ED; PUMA = Public Use Microdata Area.

A graph of the number of FrEDs by state and year is in Data Supplement S1, Figure S1. Full regression results are reported in Data Supplement S1, Table S2. The coefficients on the quarter fixed effects in Column 1 indicate that spending on EDs steadily increased throughout the sample period. Figure 2 reports adjusted estimates of the association between FrED entry into a PUMA and ED spending per enrollee. The glm regression specification with a log link implies that coefficients are interpreted as the percentage change in spending per enrollee with a one‐unit change in the explanatory variable. Texas is coded as the base state for comparison, because it contains the most FrEDs overall and the most FrEDs per PUMA. Entry of each additional FrED was associated with a 3.6 (CI = 2.4 to 4.9) percentage point increase in ED spending per enrollee in a PUMA. The state interaction terms for Florida and North Carolina were not significantly different from 0, so the association between FrED entry and spending for these two states was assumed to be similar to Texas. However, there was no change in ED spending per enrollee (−2.5, CI = −8.2 to 3.1) associated with entry of an additional FrED in Arizona.

Entry of an additional FrED was associated with a 0.18 (CI = 0.12 to 0.23) increase in the number of ED visits per 100 enrollees in Texas, Florida, and Arizona. In contrast, entry of another FrED was not associated with a reduction (−0.03, CI = −0.09 to 0.02) in utilization of EDs per 100 enrollees in North Carolina. The regression results suggested no relationship (−0.37, CI = −0.88 to 0.13) between the number of FrEDs entering a PUMA and price for Texas, Florida, and North Carolina. However, entry of an additional FrED in Arizona was associated with a 10.1 percentage point decrease (CI = −17.0 to −3.3) in the price per ED visit in Arizona. Entry of a FrED was associated with a 3.6 percentage point increase (CI = 2.5 to 4.8) in estimated out‐of‐pocket spending per beneficiary in Texas, and the magnitude was not significantly different from Texas for Florida and Arizona. However, FrED entry was associated with a 15.3 percentage point (CI = −26.8 to −3.7) reduction in out‐of‐pocket spending per enrollee in North Carolina.

In a sensitivity analysis, we limited the sample to PUMAs where 100% of the land area was located in a Metropolitan Statistical Area. The regression results remained virtually unchanged. An additional sensitivity analysis revealed that entry of an additional FrED was also associated with a 3.6 percentage point (CI = 2.3 to 5.0) increase in the insurer's amount paid per enrollee in Florida, North Carolina, and Texas. Similar to the spending estimates, FrED entry was not associated with a significant change in paid amounts by the insurer in Arizona (−5.1, CI = −11.3 to 1.0).

## Discussion

FrEDs have been touted as a means to reduce wait times and excess demand at overburdened hospital EDs.^18^, ^30^, ^31^ They have also been associated with lower inpatient hospital admission rates relative to hospital‐based EDs.^32^ However, the same sources that suggest that FrEDs could function as a substitute for hospital EDs also remark that these facilities could lead to an increase of ED visits for nonemergency conditions.^8^, ^9^

One study of a hospital ED at a tertiary care center found that patient volume fell 7.5% during a period of 3 years when two FrEDs opened nearby, while total patient volume for all three facilities combined rose 45%.^33^, ^34^ Another study found that Medicare expenditures per beneficiary were $55 higher for each FrED that entered a county between 2003 and 2009. However, this study could not isolate the relationship between FrED entry and ED spending.^35^

This study is the first we know of that measures the relationship between entry of FrEDs and overall spending on emergency services in a large sample of local markets. Our sample contains data from Arizona, Florida, North Carolina, and Texas, which include 20.4% of the U.S. population.^36^ The observed differences in spending between states were generally consistent with price differentials for an ED visit reported by the Health Care Cost Institute (HCCI). The HCCI found the price of an ED visit to be between 110 and 133% of the national average in Texas, between 100 and 110% of the national average in Arizona and North Carolina, and between 90 and 100% of the national average in Florida.^37^

In three of four states, we found a positive association between entry of a FrEDs in a PUMA and average ED spending per enrollee. Entry of an additional FrED was associated with a 3.6% increase in spending per enrollee in Texas PUMAs, and the results for Florida and North Carolina were not statistically significantly different from 3.6%. In Arizona, entry of a FrED in a PUMA was not associated with a change in spending per enrollee.

When we separated the changes in spending into parts attributable to changes in utilization versus price, there was a significant increase in utilization for Texas and Florida, but no significant change in price. For North Carolina, there was no significant change in utilization associated with FrED entry. We also found no significant increase in price associated with entry of FrEDs in North Carolina, although the magnitude of the estimate was relatively large (1.8 percentage points, CI = −4.7 to 8.4).

For Arizona, the increase in utilization was not significantly different from Texas, but there was also a statistically significant 10.1% fall in price. It is possible that FrED entry was associated with more aggressive price competition in Arizona than in other states. More FrEDs in Arizona may have become in‐network for insurers than in other states, which would have lowered prices. Unfortunately, we could not distinguish between in‐network and out‐of‐network claims in the BCBS Axis data. More light could be shed on these hypotheses with access to an All‐Payers Claims Database.^38^

Entry of a FrED in a PUMA was associated with an increase in out‐of‐pocket spending per enrollee for Texas, Florida, and Arizona, but out‐of‐pocket spending per capita dropped a remarkable 15.3 percentage points for North Carolina. Of the four states we examined, Texas is the only state that allows independent FrEDs. Previous studies have noted potentially different incentives between independent and satellite FrEDs.^4^, ^20^ Hospitals may open satellite FrEDs in the hopes of attracting more inpatient admissions, but in the three states with only satellite FrEDs, we observe different relationships between entry and out‐of‐pocket spending. We were able to confirm that all of the visits by BCBS enrollees to EDs in North Carolina were to in‐network facilities. However, some of the hospital EDs had out‐of‐network physicians providing emergency care. North Carolina patients may have shifted their ED utilization away from hospital EDs with out‐of‐network physicians and toward FrEDs with in‐network physicians, which may have reduced their payment burden.

Health insurers and policy researchers have been critical of FrEDs because of their potential for raising overall ED expenditures. The Medicare Payment Advisory Commission recommended in June 2018 to reduce Type A ED payment rates by 30% for satellite EDs that are within 6 miles of a hospital‐based ED. MedPAC's rationale was that the current Medicare payment system creates incentives for providers to treat lower‐intensity patients in the ED setting rather than in urgent care centers, which are paid less than half the Type A payment rates for ED services.^9^ If insurers and FrED owners could reach an agreement to reduce facility fees for patients with nonemergent conditions at FrEDs, patients could benefit by receiving timely and affordable access to care, while FrED operators could still earn additional revenues for filling an unmet need in the market.

One might be concerned that the increase in prevalence of high‐deductible plans^39^ and insurers’ attempts to deny claims for allegedly unnecessary visits to EDs^29^ have placed a greater burden of FrED spending on patients versus insurers. However, a sensitivity analysis indicated that both total spending per enrollee and payments solely by the insurer rose by the same amount (3.6 percentage points) with entry of a new FrED. This result suggests that the proportion of the total bill that patients are responsible for does not increase with FrED entry.

## Limitations

We mention several caveats to our analysis. Our data do not allow us to distinguish between claims filed by FrEDs versus those that came from hospital EDs. For this reason, we can only measure the association between FrED entry and overall ED spending per enrollee in a local market. Nevertheless, this analysis is much more precise than a recent study comparing the number of FrEDs in a county and total Medicare expenditures.^35^

Our analyses did not adjust for patient case‐mix severity, which has been found to be lower at FrEDs compared to hospital‐based EDs.^40^ However, our dependent variable is spending per BCBS enrollee not spending per visit. Spending per enrollee can rise even if entering FrEDs treat lower‐severity patients, as long as utilization per enrollee and the price per visit do not fall.

We aggregated ED claims based on the zip code of each enrollee because the claims lack accurate information on the provider's location. Hospitals tend to submit claims for their satellite FrEDs using the location of their hospital‐based ED. Some enrollees may visit an ED outside of their PUMA of residence. Assuming that the tendency to obtain emergency care outside an enrollee's PUMA is random across PUMAs, the association between FrED entry and the dependent variables of interest would be biased toward zero.^41^ Therefore, we may have underestimated the association between FrED entry and spending, utilization, and price.

We lack data on the number of primary care or urgent care practices by PUMA. FrEDs may have entered in areas where such facilities existed, which would legitimately increase ED utilization. However, other studies suggest that FrEDs enter in high‐income, well‐insured neighborhoods,^20^ which are the same factors that attract both primary care physicians and urgent care clinics.^42^, ^43^

Previous research found that state policies regarding FrEDs vary widely.^18^ Florida and North Carolina maintain CON regulations, while Arizona and Texas do not. North Carolina has no FrED‐specific policies, while the other states do. Other differences in rules across the states we examined are reproduced in Data Supplement S1. With multiple differences in regulations, we are unable to determine what role any specific policy played in the spending, utilization, and price changes we observed.

Patients may not have paid their portion of the allowed amount reported in the insurance claims data, which would have led to overestimated spending. However, we have no reason to believe that hospitals and FrEDs differ in their ability to collect payments from patients. If the rate of underpayment is the same for hospital EDs and FrEDs, then the percentage changes in spending per enrollee associated with FrED entry that we estimated remain the same.

The BCBS Axis limited data set had no claims data for Arizona in 2013 and no claims information for eight quarters in Florida. However, the trends in Figure 1 are consistent with an upward trend in spending for all four states throughout the sample period. These missing claims data do not bias the regression estimates, because the regressions include quarter fixed effects that account for the quarter and year in which each observation occurs.

## Conclusion

In conclusion, entry of freestanding EDs has increased local market spending on EDs in three of four states’ markets where they have entered. The increase in spending for Texas and Florida was accompanied by a rise in utilization of EDs. The increase in spending observed for North Carolina was accompanied by a relatively large but statistically insignificant price increase. Entry of freestanding EDs were associated with a decline in the price of ED visits in Arizona, an increase in utilization, and no change in spending. State policy makers and researchers should carefully track spending and utilization of emergency care as freestanding EDs disseminate to better understand their potential health benefits and cost implications for patients.

Rice University participates in the Blue Cross Blue Shield Alliance for Health Research. The Blue Cross Blue Shield Association established the Blue Cross Blue Shield Alliance for Health Research to engage leading U.S. health care researchers in collaborative efforts to use a limited data set drawn from Blue Cross Blue Shield companies to explore critical health care issues to improve the health of Americans. The Blue Cross Blue Shield Alliance for Health Research provides researchers with use of a secure data portal to access a limited data set from Blue Cross Blue Shield Axis, the largest collection of commercial insurance claims, medical professional, and cost‐of‐care information. Blue Cross Blue Shield Association is an association of independent Blue Cross Blue Shield companies.

## Supporting information


**Data Supplement S1.** Supplemental material.
