# Supplementary material for: Freestanding Emergency Department Entry and Market‐level Spending on Emergency Care
Source: Acad Emerg Med. 2019 Oct 22;26(11):1221–31. doi: 10.1111/acem.13848 (PMC6899627; doi:10.1111/acem.13848)
Supplement: Supplementary file 1 — Data Supplement S1. Supplemental material. [file ACEM-26-1221-s001.docx]

**Data Supplement S1. Supplemental material.**

| **Table S1: State Requirements of Freestanding Emergency Departments in the U.S., 2015^a^** | | | | | | | | | |
| --- | --- | --- | --- | --- | --- | --- | --- | --- | --- |
| **State** | **Number of FrEDs in 2015** | **FrED-specific policies** | **Certificate of Need** | **Hospital affiliation required** | **License Required** | **Open 24/7** | **Required to receive ambulances** | **Physician on site** | **2017 Population^b^** |
| Arizona | 6 | Y | N | N | Y | Y | N | Y | 7,048,876 |
| Florida | 23 | Y | Y | Y | N | N | N | N | 20,976,812 |
| North Carolina | 12 | N | Y | Y | N | Y | Y | Y | 10,270,800 |
| Texas | 194 | Y | N | N | Y | Y | Y | Y | 28,322,717 |
|  |  |  |  |  |  |  |  |  |  |
| ^a^All information except for the last column is obtained from Reference 16, Appendix Exhibit A1. | | | | | | | |  |  |
| ^b^Population estimates are from Reference 35. | | | |  |  |  |  |  |  |

| **Table S2. Determinants of Emergency Department Spending per Capita, Utilization, and Price by Public Use Microdata Area^d^** | | | | | | | | | |
| --- | --- | --- | --- | --- | --- | --- | --- | --- | --- |
|  |  |  | (1) |  | (2) |  | (3) |  | (4) |
| DEPENDENT VARIABLE |  |  | Spending^a^ |  | Utilization^b^ |  | Price |  | Out-of-pocket Expense^c^ |
|  |  |  |  |  |  |  |  |  |  |
| VARIABLES |  |  |  |  |  |  |  |  |  |
|  |  |  |  |  |  |  |  |  |  |
| # of FrEDs |  |  | 0.0363** |  | 0.175** |  | -0.00374 |  | 0.0363** |
| # of FrEDs * FL |  |  | 0.00668 |  | -0.028 |  | -0.0383 |  | 0.0680 |
| # of FrEDs * NC |  |  | 0.0101 |  | -0.211** |  | 0.0183 |  | -0.189** |
| # of FrEDs * AZ |  |  | -0.0618* |  | 0.012 |  | -0.0975** |  | 0.00560 |
| % Medicare |  |  | -0.0392 |  | 0.091 |  | -0.0675 |  | -0.426* |
| % Black |  |  | 0.127 |  | 0.277 |  | -0.0806 |  | 0.180 |
| % Hispanic |  |  | -0.276* |  | -0.579 |  | -0.0513 |  | -0.112 |
| % High school graduates |  |  | 0.164 |  | 0.570 |  | 0.102 |  | 0.236 |
| 2013 | Q1 |  | --- |  | --- |  | --- |  | --- |
|  | Q2 |  | 0.0356** |  | -0.006 |  | 0.0216** |  | -0.0667** |
|  | Q3 |  | 0.0547** |  | -0.051 |  | 0.0315** |  | -0.102** |
|  | Q4 |  | 0.0916** |  | 0.037 |  | 0.0352* |  | -0.0715** |
| 2014 | Q1 |  | 0.124** |  | 0.001 |  | 0.0809** |  | 0.0813** |
|  | Q2 |  | 0.170** |  | 0.090** |  | 0.0910** |  | 0.0172 |
|  | Q3 |  | 0.172** |  | 0.027 |  | 0.0962** |  | -0.0599** |
|  | Q4 |  | 0.222** |  | 0.154** |  | 0.140** |  | -0.0575** |
| 2015 | Q1 |  | 0.276** |  | 0.127** |  | 0.163** |  | 0.179** |
|  | Q2 |  | 0.339** |  | 0.180** |  | 0.198** |  | 0.113** |
|  | Q3 |  | 0.360** |  | 0.162** |  | 0.208** |  | 0.0513* |
|  | Q4 |  | 0.365** |  | 0.124* |  | 0.253** |  | -0.0101 |
| 2016 | Q1 |  | 0.436** |  | 0.273** |  | 0.285** |  | 0.221** |
|  | Q2 |  | 0.445** |  | 0.198** |  | 0.345** |  | 0.116** |
|  | Q3 |  | 0.447** |  | 0.119 |  | 0.337** |  | 0.0384 |
|  | Q4 |  | 0.454** |  | 0.113 |  | 0.328** |  | -0.0112 |
| 2017 | Q1 |  | 0.494** |  | 0.201** |  | 0.390** |  | 0.221** |
|  | Q2 |  | 0.490** |  | 0.112 |  | 0.385** |  | 0.0933** |
|  | Q3 |  | 0.467** |  | 0.037 |  | 0.385** |  | -0.0120 |
|  | Q4 |  | 0.521** |  | 0.245** |  | 0.362** |  | -0.0182 |
|  |  |  |  |  |  |  |  |  |  |
| Constant |  |  | 4.324** |  | 5.084** |  | 7.294** |  | 3.426** |
| # of Observations |  |  | 8,476 |  | 8,476 |  | 8,476 |  | 8,476 |
| Robust standard errors in parentheses, ** p<0.01, * p<0.05 | | | | | | | | | |
| ^a^The allowed amount, (paid by BCBS plus out-of-pocket payment) per BCBS member in the PUMA | | | | | | | | | |
| ^b^Number of visits per 100 beneficiaries in the PUMA | | | | | | | | | |
| ^c^Patient's estimated out-of-pocket expense per BCBS member in the PUMA | | | | | | | | | |
| ^d^Regressions also include PUMA fixed effects and interactions of each PUMA fixed effect with a linear time trend. | | | | | | | | | |
